# Quality improvement of nursing on patients with complex cerebral arteriovenous malformation undergoing hybrid surgery: a prospective single-center study

**DOI:** 10.1186/s41016-021-00240-6

**Published:** 2021-05-01

**Authors:** Dong-Hong Zhao, Rui Xue, Xiao-Rong Sun

**Affiliations:** 1 Department of Neurosurgery, Beijing Tiantan Hospital, Capital Medical University, Beijing, 100079 People’s Republic of China; 2 China National Clinical Research Center for Neurological Diseases, Beijing, People’s Republic of China; 3 Center of Stroke, Beijing Institute for Brain Disorders, Beijing, People’s Republic of China; 4 Beijing Key Laboratory of Translational Medicine for Cerebrovascular Disease, Beijing, People’s Republic of China

**Keywords:** Intensive nursing, Hybrid surgery, Arteriovenous malformation

## Abstract

**Background:**

We sought to explore an optimal clinical nursing mode following a hybrid surgery for cerebral arteriovenous malformation.

**Methods:**

Patients with complex cerebral arteriovenous malformations seen in our neurosurgery department from January 2016 to December 2017 were prospectively enrolled. The hybrid surgery protocol included “angiographic diagnosis, surgical resection, and intraoperative angiographic evaluation” and “angiographic diagnosis and embolization, surgical resection, and intraoperative angiographic evaluation”. The patients were randomly stratified into intensive care group and routine care group. After surgery, intensive or routine care was provided, and the prognosis of patients was evaluated, with a subsequent comparative analysis.

**Results:**

A total of 109 cases were divided into the routine nursing group (*n* = 54 cases) and intensive nursing group (*n* = 55 cases). There were no significant differences between the two groups in baseline data before surgery. Postoperative lung infection in the intensive nursing group was significantly less frequent than those in the routine nursing group (5.5% vs. 18.5%, *P*=0.039) with pulmonary infection and lower extremity venous thrombosis (5.5% vs. 24.1%, *P*=0.006). The average hospital stay in the intensive nursing group was 14.4 ± 5.78 days, which was significantly lower than that in the routine nursing group (19.3 ± 6.38 days, *P*=0.013). At 3 months’ follow-up after surgery, the Generic Quality of Life Inventory-74 (GQOLI-74) dimension score and GQOLI-74 total score in the enhanced group were significantly better than those in the routine nursing group (*P*=0.017 and 0.023, respectively).

**Conclusions:**

Intensive postoperative nursing can improve the safety of patients after hybrid surgery, reduce the postoperative complications and the average length of hospital stay, and improve the quality of life of patients.

## Background

Complex cerebral arteriovenous malformations are cerebral arteriovenous malformation with a giant size, complex angioarchitechture, and involvement of eloquent structures. The condition is often associated with multidisciplinary treatment strategies that are difficult to choose, with a high cost for treatment and a higher risk of postoperative complication after both endovascular and open surgery procedures. The earliest hybrid surgery can be traced back to vascular surgery carried out in the 1960s, having been rapidly developed and applied in heart surgery since then. In recent years, neurosurgery has gradually begun to use hybrid surgery technology [1, 2]. At present, complex operation is mainly used in the treatment of complex cerebral arteriovenous malformations [3]. Before the surgery, the main blood supply branches are blocked or embolized, the branches in the malformation are reduced, and the bleeding in the separation is limited [4]. The main steps of the hybrid operation for the treatment of cerebral arteriovenous malformations include (1) preoperative angiography of the whole cerebral artery, (2) preoperative embolization, (3) angiography reexamination of embolization results, (4) craniotomy for the removal of vascular malformations, (5) angiography reexamination of the surgical results, and (6) adequate hemostasis followed by craniotomy. The blood supply of complex cerebral arteriovenous malformations is abundant, which results in chronic hypoperfusion of the surrounding brain tissue. After resection or embolization of arteriovenous malformations, the normal brain tissue that has been adapted to low perfusion appears damaged in terms of its ability to self-regulate changes in blood pressure and carbon dioxide. In the normal range, perfusion pressure will still cause brain congestion, edema, and even hemorrhage [5]. The poor postoperative state of these patients results in an increased nursing difficulty, so it is necessary to improve the level of nursing care, to enhance patients’ and their families’ knowledge and understanding of disease [6], and to help patients through the postoperative risk period [7]. This study investigated the method and value of enhanced perioperative nursing care for patients with hybrid surgery for complex cerebral arteriovenous malformations.

## Methods

The study protocol was approved by the Institutional Review Board of our hospital. Written informed consent was obtained from all participants and their guardians at admission.

### Study population and sample size

We assumed an average of complication rate of 20% with routine nursing after hybrid surgery for complex cerebral AVM and 8% with intensive nursing. Minimum detectable odds ratios (OR) were computed at 80% power and a 5% false-positive rate using the PS program. The estimated sample size was 43. From January 2016 to December 2017, 109 patients with complex cerebral arteriovenous malformations were enrolled in the study. Their baseline data, nursing data, surgical information, and clinical follow-up data were collected.

### Setup

All of the included patients had complicated cerebral arteriovenous malformations diagnosed by imaging and were treated by hybrid operation. After admission, patients were randomly stratified into a routine nursing group and intensive nursing group with computer randomization.

The following protocols were included in routine nursing: raise the head of the bed 15–30° after operation to promote venous return; keep patient lying in bed to rest, monitor vital signs, strictly monitor blood pressure, and maintain high pressure below 130 mmHg; observe the healing of the head wound and femoral artery puncture point to avoid infection; guide patients to defecate, avoid mental stimulation, avoid violent cough, and keep defecation unobstructed; and, ultimately, recover good condition in patients. In the case of severe headache, painkillers are appropriate after elucidating the cause. Before discharge, it is necessary to provide discharge guidance, inform the patients of rehabilitation exercise methods, and instruct them to come to the hospital for reexamination regularly.

Separately, in addition to those reported above in routine nursing, intensive nursing also included the following protocols:

1. Setup of an intensive nursing team, composed of one deputy chief nurse, two chief nurses, and one responsible nurse. All of the members were trained before the experiment on knowledge related to hybrid operation, arteriovenous malformation, rehabilitation training, quality of life scale use, visiting the operating room, and watching the operation video. The training time was 1 month, and all of the members were trained by specialists at our hospital. After training, the appropriate members to be responsible for all of the aspects of the experiment, such as the evaluation of patients’ condition before operation, psychological guidance before and after operation, evaluation and rehabilitation guidance of postoperative dysfunction, and follow-up of postoperative quality of life scale, were selected.
2. Preoperative full evaluation and preparation of related items. The nurse in charge of the 2 days before the operation discussed the patient’s condition, operation plan, and team division in detail. Nursing cooperation not only needs to involve the full understanding of the patient’s condition and operation plan but also may have to pursue further communication with the doctor as to what approach to take in the operation and related complications that may occur after the operation. In the operation of vascular malformation [8], for example, requirements included preparing the thrombus elastic pump, air pump bed, and expectorant. Before surgery, the patients’ limb movement and muscle strength are quantified, and the symptoms of neurological deficit are evaluated strictly, which is convenient for comparison after operation. Patients with cerebrovascular diseases often have coagulation dysfunction after a long period of surgery time. It is necessary to know the coagulation function of patients to prevent thrombosis after operation. The skin in the groin and head operation areas should be cleaned, prepared, and protected, and postoperative pressure injuries should be prevented.
3. Preoperative psychological guidance to improve patients’ and their families’ confidence in the operation. The hybrid operation of cerebral arteriovenous malformation could be complex, the implementation is difficult and takes a long time, and the patients and their families can be prone to anxiety and fear. In view of the above problems, the responsible nurses should focus on humanistic care. At the right time before the operation, a nurse would explain to the patients and their families by mainly informing about the process, method, duration, risk of the operation, and the health education knowledge related to the hybrid operation so that fear caused by misunderstanding can be eliminated and the potential postoperative complications, psychological problems, impact on the quality of life, and the nursing measures to be taken during the perioperative operation can be clarified. A nurse would also emphasize the length of operation, reduce the waiting anxiety of patients’ families, and guide patients’ families to cooperate with medical staff to do a good job in ensuring patients pacification [5]. For psychological goal-setting, the specific methods include changing the patients’ cognition of the disease and improving their confidence of achieving a cure. Steps to take include the following: (1) through the explanation of disease-related knowledge by nurses, reduce the patients’ high expectations of treatment goals and rational cognition; (2) through the explanation of the operation process, inform about the advantages of the hybrid operation, the possible normal conditions and related complications after the operation, and the nursing measures taken; and (3) the nursing goal should be made into quantifiable and executable actions. To change patient thinking, specific methods include (1) face up to emotion, pursue reasonable expression, provide a safe and comfortable environment for patients, improve nurse language skill, present an amicable attitude, and keep patients in a happy mood—in short, nurses should guide patients to vent reasonably and enhance their confidence in overcoming the disease. Further, (2) responsible nurses should organize on-site discussions of successful patients, exchange successful experiences with each other, and provide psychological support to each other. Third, (3) communication is the most important—nurses should pay close attention to the psychological state of patients, aiming at addressing the problems that arise at different periods.
4. Intensive nursing after operation. The difficulty inherent in a hybrid operation to prevent thrombus lies in not only preventing hemorrhage in the traditional operation but also avoiding thrombus formation during the endovascular intervention. After the operation, patients should be confirmed to have no thrombus by venous ultrasound of both lower limbs, while routine testing should indicate the D-dimer is in the normal range. After the operation, patients can be given elastic socks and the thrombus elastic pump used twice a day according to the doctor’s instructions, each time for 2 h. When the patient’s body allows, we advocate them to walk on the ground as early as possible to promote physical recovery. The long time period of combination operations is a risk factor for infection. During and after the operation, aseptic operation should be strictly carried out to avoid cross-infection. At the same time, the patient’s temperature and wound condition should be strictly monitored and anti-infection treatment should be implemented at all times. Neurological dysfunction is a common postoperative complication in the operation of arteriovenous malformation, especially in the functional area. Therefore, after the operation, the neurological status of patients should be strictly evaluated and compared with that before the operation. In the case of new dysfunction, we should assign special members to give rehabilitation guidance three times a day. At the same time, we should supervise the operation of patients and their families. For patients with serious postoperative complications, the research members in charge of psychological counseling should provide psychological counseling to patients and their families and adjust the number of counseling sessions according to the satisfaction of patients.

### Evaluation indexes and approaches

Clinical data, including AVM rupture, S-M grading, whether the lesion is located in the functional area, deep vein drainage, AVM size, operation time, and postoperative complications, were obtained through chart review. DSA was reexamined in all of the patients with complex arteriovenous malformations to determine whether there were residual lesions. Patients were followed up by telephone. The follow-up included the outcome after treatment of the patients’ original symptoms, any new cerebrovascular events, and quality of life assessment using parts of the Generic Quality of Life Inventory-74 (GQOLI-74) questionnaire (i.e., GQOLI-74 dimension score, GQOLI-74 factor score, SAS emotional state score, SDS emotional state score, and depression degree). The quality of life assessment was completed by the same nurse after training. The first follow-up time was 3 months.

### Statistical analysis

The statistical software used in this study was SPSS Statistics version 22.0 (IBM Corp., Armonk, NY, USA). The measurement data of normal distribution are expressed as means ± standard deviations (SD) or as frequencies and percentages (%). Chi-squared analysis was used to compare the rates between the two groups. The continuous variables were compared using *t*-test for normal distribution data or Wilcoxon rank-sum test for non-normal distribution data. The distribution of data was tested using *F* test. A *p* value of less than 0.05 was considered to be statistically significant.

## Results

### Baseline data of the study population

From January 2016 to December 2017, 109 patients with complex cerebral arteriovenous malformations were treated in our ward by hybrid surgery, including 60 males and 49 females aged 9 to 54 years (median age 26 years). Before their operations, all of the patients were confirmed as having giant arteriovenous malformation by imaging examination. There were clinical symptoms of different degrees noted in the perioperative period: 87 patients (79.8%) had headache, 34 patients (31.2%) had epilepsy, and 68 patients (62.4%) had bleeding. Other clinical baseline data included AVM rupture, S-M grading, whether the lesion is located in the functional area, deep vein drainage, and AVM size (Table 1). There were no significant differences in baseline characteristics between the two groups.

**Table 1:** Baseline of patients with complex cerebral arteriovenous malformation

	Ordinary nursing (*n* = 54)	Intensive nursing (*n* = 55)	*p* value
Gender (male, %)	29 (53.7)	31 (56.4)	0.780
Age (mean ± SD)	27.1±12.29	24.4±13.91	0.284
AVM rupture (yes, %)	35 (64.8)	33 (60.0)	0.604
S-M grading score (mean ± SD)	2.3 ± 1.08	2.7 ± 0.89	0.578
Eloquent (yes, %)	22 (40.7)	25 (45.5)	0.619
Deep venous drainage (yes, %)	13 (24.1)	16 (29.1)	0.553
AVM size (cm, %)			0.599
≤3	22 (40.7)	19 (34.5)	
3–6	29 (53.7)	31 (56.4)	
≥6	3 (5.6)	5 (9.1)	
mRS at admission (*n*, %)			0.479
0–2	51 (94.4)	50 (90.9)	
3–4	3 (5.6)	5 (9.1)	

### Comparison of perioperative conditions between the two groups

All of the patient operations were performed successfully, and symptoms and signs of cerebral ischemia improved significantly after surgery. Symptoms of high intracranial pressure, such as short-term headache, dizziness, nausea, and vomiting, occurred in 47 patients after operation, but all cases improved after 3–5 days of intravenous administration of mannitol. There were no cases of stroke, cerebral hemorrhage, or death within 2 weeks after operation. As compared with preoperative evaluation, the function of four patients (3.7%) was improved, 78 patients (71.6%) showed no significant change, and 27 patients (24.8%) presented decreased function. There was no statistical difference in the operation time between the two groups, while the incidence rates of postoperative pulmonary infection and lower extremity venous thrombosis in the intensive care group were significantly lower than that in the routine care group. The average hospital stay in the routine care group was 19.3 ± 6.38 days, while the average hospital stay in the intensive care group was 14.4 ± 5.78 days, with a significant statistical difference between the two groups (Table 2).

**Table 2:** Comparison of perioperative conditions between the ordinary nursing and intensive nursing groups

	Ordinary nursing (*n* = 54)	Intensive nursing (*n* = 55)	*p* value
Total resection (yes, %)	50 (92.6)	48 (87.3)	0.357
Surgery time (h, mean ± SD)	7.3 ± 2.04	7.8 ± 1.12	0.774
Postoperative complication (*n*, %)			
New onset intracranial hemorrhage	3 (5.6)	2 (3.6)	0.632
New onset intracranial ischemia	5 (9.3)	7 (12.7)	0.563
Neurological dysfunction	10 (18.5)	9 (16.4)	0.726
Intracranial infection	4 (7.4)	6 (10.9)	0.527
Lung infection	10 (18.5)	3 (5.5)	0.039
Deep venous thrombosis	13 (24.1)	3 (5.5)	0.006
Hospital stay (days, mean ± SD)	19.3 ± 6.38	14.4 ± 5.78	0.013
mRS at discharge (*n*, %)			0.283
0–2	43 (79.6)	48 (87.3)	
3–4	11 (20.4)	7 (12.7)	

### Comparison of quality of life at 3 months after surgery between the two groups (Table 3)

The average length of time to the first follow-up review was 3.2 months. The telephone follow-up evaluation found that the psychological function and emotional state of the patients in the intensive care group at 3 months after operation were significantly better in comparison with the routine care group.

**Table 3:** Comparison of quality of life at 3 months after operation between the ordinary nursing and intensive nursing groups

	Ordinary nursing (*n* = 54, mean ± SD)	Intensive nursing (*n* = 55, mean ± SD)	*p* value
GQOLI-74 dimension score	284.2 ± 70.34	311.2 ± 59.64	0.017
Physical function	74.8 ± 18.79	79.3 ± 16.04	0.172
Social function	77.5 ± 18.61	85.3 ± 15.92	0.043
Psychological function	79.6 ± 18.68	94.4 ± 15.88	0.027
Material life	62.2 ± 14.63	65.5 ± 12.64	0.205
GQOLI-74 factor score	298.7 ± 53.72	312.2 ± 45.54	0.163
GQOLI-74 total score	66.9 ± 21.98	75.27 ± 18.64	0.023
SAS emotional status score	40.18 ± 12.6	35.6 ± 12.79	0.043
SDS emotional status score	56.9 ± 11.78	50.8 ± 11.18	0.054
Depression severity	1.05 ± 0.88	1.00 ± 0.86	0.739

## Discussion

It has been reported that the incidence of cerebral arteriovenous malformation is about one in 100,000, and the most common symptom of such is intracranial hemorrhage. It is necessary to comprehensively evaluate the risk factors and status of patients with cerebral arteriovenous malformation bleeding in the clinic and to actively deal with the significant risk factor of bleeding so as to reduce the risk of disability and death caused by such. With the development of new technology, more and more scholars have considered multidisciplinary and comprehensive treatment in the hybrid operating room [9]. In order to improve the postoperative quality of life and reduce the frequency of complications, we should take active measures to effectively reduce postoperative complications caused by improper nursing, effectively shorten the hospitalization time, and limit hospitalization expenses [10]. This study explored intensive nursing methods to help patients recover from their poor state as soon as possible and observed an improved nursing effect. A hybrid operation approach can effectively evaluate the effects of operation especially involving cerebral arteriovenous malformations in large size and functional areas and remove the residual lesions of cerebral arteriovenous malformations under precise positioning so as to reduce the surgical injury and unnecessary brain tissue removal and better protect and retain the function of brain tissue [11]. Combined with the contents of existing medical literature reports, the hybrid operation of cerebral arteriovenous malformation is expected to achieve the goal of cure [12]. Hybrid operation technology can evaluate the curative effect in real time during the operation of cerebral arteriovenous malformation, guide the operation implementation, avoid possible risks associated with patients’ transfer between related disciplines and multiple anesthesia inductions, and improve the safety and cure rate of the operation [13]. In our patient cohort, the symptoms of cerebral hemorrhage and ischemia were significantly improved, and there were no obvious perioperative complications. There was occasionally blurred vision in two patients, which gradually improved during the follow-up period.

The treatment goal of hybrid surgery is to remove the foci and improve the neurological function, while the goal of nursing is to prevent complications and improve patients’ psychological health and understanding of postoperative conditions [14, 15], thereby improve the quality of life of patients, and reduce the length and the cost of the hospital stay [16]. The proposed intensive nursing protocol of 109 cases in this group is effective and indicates that a specific trained nursing team, intensive nursing with both psychological and clinical care for patients, and more integrative nursing after open and endovascular intervention would contribute to improved quality of life and lower postoperative complication in patients with hybrid surgery. Patients with complex AVMs are usually in a poor perioperative state [17] and more often than not have psychological problem and chronic headache [18]. During the treatment period, they are prone to have negative emotions, such as anxiety and depression due to excessive worry, which can aggravate their condition and reduce the treatment effect [16]. Therefore, it is very important to introduce effective nursing interventions for these patients. The main purpose of bedside rounds is to provide patients with the best treatment, nursing, and human care. During the period of disease treatment, the clinical application of personalized bedside rounds can improve the working efficiency and nursing quality of medical staff, enhance the communication between nurses and patients, get patients to have a sense of trust in nursing staff, and significantly improve patients’ treatment compliance [15]. In this study, the individualized nursing mode was used to intervene in cases of patients with complex intracranial arteriovenous malformation. Through bedside ward rounds, the rights and responsibilities of nurses can be clarified, and the continuity of nursing work can be enhanced. Further, such can ensure that the responsible nurses transfer feedback about patients’ conditions to the attending physicians in time, and then, through information exchange, the responsible nurses and the attending physicians can have a comprehensive understanding of the patients’ condition and develop the formulated nursing prescription. The case has pertinence; the responsible nurse and the attending physician explain the disease-related knowledge and nursing intervention items patiently for the patients from different angles, which can help the patients gain more health knowledge, improve their self-confidence, significantly improve the quality of life of the patients, and accelerate their recovery progress. The results of this study showed that the scores of GQOLI-74 were better than those of the control group (*p* < 0.05), suggesting that individualized nursing intervention can effectively regulate the negative emotions of patients, significantly increase their disease awareness. Therefore, a personalized nursing mode in the nursing of complex cerebrovascular malformation can significantly improve the treatment compliance of patients, effectively lessen their negative emotions, and increase their postoperative quality of life.

In addition to an improved nursing mode, a specific training for the nursing team would also be important to provide more integrative care for AVM patients with hybrid surgery. The responsible nurses should fully understand the preoperative examination, positive signs, and the expected psychological effects on patients; explain the relevant disease knowledge, operation methods, and operation methods; consult the literature; and take active and effective nursing measures based on the actual situation of patients by applying nursing procedures. Every day, the responsible nurses provide the patients with personalized nursing services that integrate diagnosis and treatment as well as rehabilitation. The responsible nurses can express their own opinions on the nursing problems of the patients during the morning shift handover, discuss with each other to determine the nursing problems according to the factors that may affect patients’ conditions, and help colleagues to judge how to approach certain cases. To deal with the actual problems of patients and help patients build up confidence so that they closely cooperate with the treatment and nursing, nurses must take nursing rounds seriously. The responsible nurse turns over the shift every morning to carry out routine rounds with the director and the attending physician. The responsible nurse feeds back the patient’s condition to the attending physician, including vital signs, eating habits, defecation, limbs, language, and patient activities. According to the doctor’s guidance, nurses should adjust the observation focus; monitor various laboratory indicators; and develop personalized diagnosis and treatment, rehabilitation, and nursing plans for the patients. Nurses should also offer timely health education. The responsible nurse can explain the pathogenesis, symptoms and diagnosis, and treatment methods of intracranial complex arteriovenous malformation to the affected patients in detail. The responsible nurse must also inform patients of relevant nursing measures and daily precautions so as to improve the patients’ cognition of the disease, establish their confidence to overcome the disease, and fully promote their cooperation with the treatment and various nursing measures. In this study, the nurses in charge asked the patients about their knowledge of disease and health education by means of cross-examination and strengthened their guidance of the patients with problems. Nurses should also pay attention to psychological nursing, striving to fully understand the psychological status of patients. Because of the large and complex trauma of the operation, the causes of negative emotions should be analyzed in-depth. Nurses can eliminate the bad emotions of patients through psychological counseling, psychological suggestion, peer education, and other ways. Through the dynamic analysis of patients’ psychological situation, in order to implement effective psychological intervention in time so as not to affect the recovery progress of patients, nurses should listen to physicians where appropriate. For patients who complain of pain after surgery, the nurse in charge could use the Changhai pain ruler to evaluate the position, degree, scope, and nature of the pain to address however appropriate. If necessary, the nurse should follow the doctor’s advice to intervene in the pain and observe the effect and reaction after using the medicine. Nurses can also implement diet intervention. After the operation in this study, the nutrition department was invited to adjust the diet plan according to the patient’s eating habits, weight, physical index, and various laboratory indicators. Nurses can act to guide patients to eat high-protein, high-vitamin, and iron-rich food to balance their diet and increase body resistance and introduce nasal feeding fluid to patients with poor eating habits. Finally, nurses should strictly prevent infection by implementing the validity isolation system, carefully carrying out hand hygiene before and after the operation and patient interactions; restrict visits from family members; prevent cross-infection; monitor the patient’s body temperature; elucidate signs of intracranial infection; and timely follow the doctor’s instructions to give interventions. Therefore, a more integrative training and perioperative care could enhance the whole recovery of patients, properly deal with multidisciplinary postoperative problems, and thereby reduce the postoperative complication rate, especially the pulmonary infection and DVT. Although there was only a trend towards lower expenditure after intensive nursing (mean total cost, 0.134 million RMB in routine nursing group vs. 0.128 million RMB in intensive nursing group), our study showed that the average hospital stay of intensive care patients was significantly reduced than the routine care, indicating the intervention value of intensive care.

## Conclusions

In summary, patients with cerebral arteriovenous malformation may not be in good condition or have multidisciplinary postoperative complication after hybrid operation, requiring an improvement of the nursing mode and a more specific trained nursing team. Integrative nursing measures and care would be helpful to enhance these patients’ recovery.

## Data Availability

All data generated or analyzed during this study are included in this published article. Some or all data, models, or code generated or used during the study are available from the corresponding author by request.
